# Bridging primary and palliative care: A cross-sectional study of the relationship between perceived access to family physicians and caregiver burden

**DOI:** 10.1097/MD.0000000000051115

**Published:** 2026-10-16

**Authors:** Emre Vuraloglu

**Affiliations:** a Department of Family Medicine, Ondokuz Mayis University, Samsun, Turkey.

**Keywords:** caregiver burden, cross-sectional studies, palliative care, primary health care

## Abstract

This study aimed to examine the association between access to family physicians and caregiver burden among individuals providing care for terminal-stage cancer patients receiving palliative care. This cross-sectional study was conducted in the palliative care unit of a tertiary-level training and research hospital in Turkey between October and December 2025. Data were collected using the Zarit Burden Interview and the Perceived Access to Health Care Questionnaire. The relationships between variables were analyzed using Spearman correlation and multivariable linear regression analyses. Daily caregiving hours (*P* < .001) and total duration of caregiving (*P* < .001) showed a positive correlation with caregiver burden. A negative correlation was found between the overall perceived access to family physicians score and caregiver burden (ρ = −0.320, *P* < .001). In multivariable analysis, daily caregiving hours (β = 0.606, *P* < .001) and total caregiving duration (β = 0.377, *P* < .001) were identified as independent predictors increasing caregiver burden, whereas the affordability subdimension of the Perceived Access to Health Care Questionnaire demonstrated a protective effect (β = −0.685, *P* = .021). Caregiver burden increased with longer daily caregiving hours and total duration of care, whereas higher overall perceived access to family physicians was associated with lower caregiver burden. These findings suggest that strengthening the integration of primary care with palliative care may play a crucial role in alleviating caregiver burden. The study provides an original contribution to the international literature on primary care-based palliative care integration, supported by empirical data from Turkey.

## 1. Introduction

The World Health Organization defines palliative care as a holistic approach aimed at improving the quality of life of individuals and their families facing life-threatening illnesses.^[1]^ This approach not only focuses on the control of the patient’s symptoms but also encompasses the physical, psychological, and social support of family members who play an active role in the caregiving process.^[1]^

Individuals who provide care for palliative care patients face intense responsibilities as they strive to maintain their own daily lives and personal obligations while assisting patients with needs such as personal care, nutrition, and symptom management.^[2]^ This process often results in physical exhaustion, psychological strain, financial difficulties, and restrictions in social life.^[3–5]^ In particular, among caregivers of terminal-stage cancer patients, challenges in symptom management lead to significant physical, psychosocial, and economic consequences, ultimately contributing to increased caregiver burden.^[6]^ This burden may negatively affect the caregivers’ overall quality of life.^[6,7]^

Primary care services play a crucial supportive role in the palliative care process through their principles of accessibility and continuity.^[8,9]^ Family physicians can play a critical role in the palliative care process, as they are the most accessible healthcare professionals within the community, establish long-term relationships with patients and their families, and facilitate communication and coordination with other healthcare services.^[10]^ However, the relationship between caregivers’ perceived access to family physicians and the level of caregiver burden they experience has not been adequately investigated.^[11]^ The study aimed to examine the relationship between caregiver burden and perceived access to family physicians among caregivers of terminal-stage palliative care patients. The findings of this research are expected to contribute to strengthening the integration between primary and palliative care and to help build a bridge between these 2 levels of care.

## 2. Materials and methods

### 2.1. Study design and sample

This research was designed as a cross-sectional observational study. It was conducted between October and December 2025 in the palliative care unit of Kirşehir Training and Research Hospital in Turkey. The study included primary caregivers of adult patients diagnosed with terminal-stage malignancy who were receiving treatment in the palliative care unit. Inclusion criteria required caregivers to be between 18 and 65 years of age and to have been actively providing care for at least 1 month. Caregivers of patients requiring palliative care for nonmalignant conditions were excluded to minimize heterogeneity in caregiver burden and access to services. In addition, caregivers with any psychiatric or chronic illness were excluded to reduce potential confounding effects, as such conditions could directly influence caregiver burden scores. During the study period, all caregivers who met the eligibility criteria and agreed to participate were consecutively included in the study, resulting in a final sample of 120 participants. A power analysis was performed using a significance level of 0.05 and 80% power, indicating that the final sample size of 120 participants was adequate.

### 2.2. Data collection

Individuals who were primary caregivers of palliative care patients and met the inclusion criteria were informed face-to-face by the researcher and voluntarily enrolled in the study. Written informed consent was obtained from all participants through an informed consent form. Subsequently, caregivers’ sociodemographic characteristics, including age, gender, education level, relationship to the patient, total duration of caregiving, and average daily caregiving hours, were recorded. Afterward, all participants completed 2 instruments that had been validated and reliability-tested in Turkish: the Zarit Burden Interview (ZBI) and the Perceived Access to Health Care Questionnaire (PAHCQ).^[12,13]^ The ZBI was developed to assess the burden experienced by caregivers and consists of 22 Likert-type items scored from 0 to 4.^[12]^ Higher total scores indicate a greater level of caregiver burden. The PAHCQ is a 23-item, 5-point Likert-type instrument designed to measure individuals’ perceptions of access to health services.^[13]^ The scale consists of 4 subdimensions: accessibility, acceptability, accommodation, and affordability. In addition, an overall access score is calculated as the mean of all items. Higher total scores reflect better perceived access to health services. In this study, the terms “health professional” and “healthcare provider” used in the original scale were linguistically adapted to “family physician,” reflecting the participants’ primary source of healthcare. The modification was limited to replacing the generic terms “health professional” and “healthcare provider” with “family physician”; no changes were made to the items, response options, scoring system, or subdimension structure.

### 2.3. Data analysis

Statistical analyses were performed using SPSS version 26.0 (IBM Corp.). Descriptive statistics were presented as mean ± standard deviation and percentages. Various statistical methods were applied to examine the relationships between caregiver burden and sociodemographic variables. The relationships between continuous variables (age, total duration of caregiving, and daily caregiving hours) and the total ZBI score were assessed using Spearman correlation analysis. For categorical variables, Mann–Whitney *U* tests were used for two-group comparisons (e.g., gender), and Kruskal–Wallis tests were used for comparisons among more than 2 groups (e.g., education level, relationship to the patient). The bivariate associations between caregiver burden and perceived access to family physicians were also evaluated using Spearman correlation. This analysis provided insight into the direction and strength of the linear relationships among the variables. Subsequently, multivariable linear regression analysis was performed, with the total ZBI score as the dependent variable. The subdimensions of the PAHCQ scale and sociodemographic variables (age, gender, education level, total duration of caregiving, and daily caregiving hours) were included as independent variables to identify factors that independently predicted caregiver burden after controlling for potential confounders. A *P* value of <.05 was considered statistically significant.

### 2.4. Ethical consideration

This study was conducted in accordance with the Declaration of Helsinki and approved by the Ahi Evran University Scientific Research Ethics Committee (decision no: 2025-15/196, date: October 07, 2025). Written informed consent was obtained from all participants prior to their inclusion in the study.

## 3. Results

### 3.1. Descriptive statistics

A total of 120 caregivers were included in the study. The mean age of the participants was 42.7 ± 11.3 years, with 54.2% females and 45.8% males. Regarding educational level, 21.7% had primary education, 39.2% secondary education, 33.3% university degrees, and 5.8% postgraduate degrees. In terms of relationship to the patient, 53.3% were spouses, 26.7% were children, and 20.0% were other relatives or close contacts.

The mean total duration of caregiving was 9.4 ± 3.7 months, and the mean daily caregiving time was 12.9 ± 5.0 hours. The mean ZBI total score was 40.8 ± 4.6. The mean total score of the PAHCQ access was 3.04 ± 0.28, with subdimension means of accessibility: 3.20 ± 0.54, acceptability: 2.91 ± 0.50, accommodation: 3.40 ± 0.50, and affordability: 2.63 ± 0.62. Detailed information on the participants’ sociodemographic characteristics and scale scores is presented in Table 1.

**Table 1:** Sociodemographic characteristics of the participants and ZBI total score and PAHCQ overall access score.

n = 120	Mean ± standard deviation	n (%)
Age (yr)	42.7 ± 11.3	
Total duration of caregiving (mo)	9.4 ± 3.7	
Daily caregiving time (h)	12.9 ± 5.0	
Gender		
1. Female		65 (54.2)
2. Male		55 (45.8)
Educational level		
1. Primary education		26 (21.7)
2. Secondary education		47 (39.2)
3. University degrees		40 (33.3)
4. Postgraduate degrees		7 (5.8)
Relationship to the patient		
1. Spouses		64 (53.3)
2. Children		32 (26.7)
3. Other relatives or close contacts		24 (20.0)
ZBI total score	40.8 ± 4.6	
PAHCQ (overall access score)	3.04 ± 0.28	

PAHCQ = Perceived Access to Health Care Questionnaire, ZBI = Zarit Burden Interview.

### 3.2. Effect of sociodemographic variables on caregiver burden

The relationship between sociodemographic variables and caregiver burden is presented in Table 2. According to the results of the Mann–Whitney *U* test, there was no significant difference in caregiver burden between female and male caregivers (*U* = 1587.0, *P* = .290). According to the results of the Kruskal–Wallis test, there was no significant difference for educational level (*H* = 7.37, *P* = .061). Similarly, no significant difference was found according to the type of relationship with the patient (*H* = 0.03, *P* = .987).

**Table 2:** Relationship between sociodemographic variables and caregiver burden.

Variables examined in relation to caregiver burden	Test	Statistic	*P* value
Gender	Mann–Whitney *U*	1587.0	.290
Education level	Kruskal–Wallis *H*	7.37	.061
Relationship to the patient	Kruskal–Wallis *H*	0.03	.987
Total duration of caregiving (mo)	Spearman ρ	0.913	<.001
Daily caregiving hours (h)	Spearman ρ	0.927	<.001
Age (yr)	Spearman ρ	−0.109	.236

The correlations between the ZBI score and caregiving variables were analyzed using Spearman correlation. A positive correlation was found between the ZBI score and daily caregiving hours (ρ = 0.927, *P* < .001). Likewise, a positive correlation was observed between the total duration of caregiving and the ZBI score (ρ = 0.913, *P* < .001). However, no significant relationship was found between caregiver age and the ZBI score (ρ = −0.109, *P* = .236).

### 3.3. Relationship between caregiver burden and perceived access to family physicians

The relationship between caregiver burden and perceived access to family physicians was evaluated using Spearman correlation, as presented in Table 3. A moderate negative correlation was observed between the overall PAHCQ access score and the ZBI score (ρ = –0.320, *P* < .001). Subdimension analyses revealed significant negative correlations between ZBI and acceptability (ρ = –0.187, *P* = .041), accommodation (ρ = –0.190, *P* = .038), and affordability (ρ = –0.194, *P* = .034). However, the accessibility subdimension did not reach statistical significance (ρ = –0.151, *P* = .101).

**Table 3:** Relationship between caregiver burden and perceived access to family physicians.

Variables	ρ (Spearman)	*P* value
PAHCQ (overall access score)	−0.320	<.001
Accessibility	−0.151	.101
Acceptability	−0.187	.041
Accommodation	−0.190	.038
Affordability	−0.194	.034

PAHCQ = Perceived Access to Health Care Questionnaire.

In multivariable analyses using the ordinary least squares linear regression method, the primary model incorporating PAHCQ subdimensions and sociodemographic variables showed a high level of fit (*R*^2^ = 0.849). No evidence of problematic multicollinearity was observed, with variance inflation factor values ranging from 1.08 to 3.65, all below the commonly accepted threshold of 5. Independent predictors of increased caregiver burden were daily caregiving hours (β = 0.606, *P* < .001) and total caregiving duration (β = 0.377, *P* < .001). Additionally, the affordability subdimension of the PAHCQ demonstrated an independent protective effect, indicating that higher affordability scores were associated with lower caregiver burden (β = –0.685, *P* = .021). Other subdimensions and demographic variables were not statistically significant.

## 4. Discussion

In this study, caregiver burden was found to increase with longer daily caregiving hours and total caregiving duration, while higher overall perceived access to family physicians was associated with lower caregiver burden. This relationship was particularly evident in the affordability subdimension of access.

The independent association between the affordability dimension and lower caregiver burden may be particularly relevant in the Turkish healthcare context. In Turkey, the family medicine model was introduced to improve access to primary healthcare and reduce household healthcare expenditures, and previous evidence has shown that the program was associated with lower expenditures for physician visits and prescription medications.^[14]^ In the present study, the affordability dimension also reflects practical aspects of access, including ease of obtaining appointments, acceptable waiting times, and consideration of individuals’ social and economic circumstances. These factors may reduce the practical and financial difficulties experienced by caregivers when seeking healthcare and partly contribute to lower caregiver burden. However, this interpretation should be considered cautiously, as healthcare expenditures and access-related barriers were not directly assessed in this study.

Several studies in the literature have examined the factors influencing caregiver burden.^[15–19]^ In a study published by Zhu et al, a high level of caregiver burden was identified among caregivers of cancer patients receiving palliative care. The study demonstrated that daily caregiving duration and the caregiver’s psychological distress (such as anxiety and depression) were significant determinants of caregiver burden.^[17]^ In the study conducted by Chan et al, caregiver burden and associated psychosocial factors were examined among family members providing care for frail older adults with multimorbidity. The study revealed that caregiver burden was highly prevalent and emphasized that social support and the caregiver’s perceived competence play a critical role in reducing this burden.^[16]^ In addition, the study identified caregiving duration as a significant factor associated with caregiver burden.^[16]^ In a study conducted by Quadir et al in Bangladesh, caregiver burden among individuals providing care for advanced-stage cancer patients was reported to be particularly associated with the nature of the caregiver–patient relationship and the caregiver’s level of awareness regarding the patient’s prognosis.^[18]^ In another study from the literature, Ali et al evaluated caregiver burden and its associated factors among family members providing care for cancer patients.^[20]^ Being female, having longer daily caregiving hours, a history of the patient’s prior hospitalizations, and reduced sleep duration of the caregiver were all found to be significantly associated with increased caregiver burden. These findings indicate that a substantial proportion of caregivers of cancer patients experience severe physical and psychosocial challenges, highlighting the necessity of integrating caregiver support into routine health practices within oncology units.^[20]^ In our study, consistent with findings in the literature, total caregiving duration and daily caregiving hours were identified as independent factors that increased caregiver burden. However, caregiver age, gender, and relationship to the patient were not found to have a significant effect on caregiver burden. Possible explanations for these results include the fact that our sample consisted solely of adults aged 18 to 65 years and that caregivers with chronic or psychiatric conditions were excluded according to the study criteria. Furthermore, in some societies, the nature of the caregiver–patient relationship is shaped by cultural norms and family roles. Indeed, Quadir et al emphasized that in Bangladesh, caregivers often feel culturally obligated to assume the responsibility of caring for sick relatives and therefore frequently refrain from expressing their own difficulties.^[18]^ This finding indicates that caregiver burden is closely related not only to individual factors but also to the broader cultural context.

Moreover, inadequate communication between healthcare professionals and caregivers has been identified in the literature as one of the primary factors contributing to increased caregiver burden.^[21]^ This situation, particularly in the context of palliative care, increases uncertainty and anxiety in both patients and caregivers, negatively affecting adherence to treatment and care processes. Effective communication is not limited to the transfer of information; it also encompasses empathy, understanding, and trust. The role of family physicians is especially critical in this regard. Through their long-term relationships with patients and families, family physicians are well acquainted with their social context and living conditions. Therefore, their use of an empathetic, clear, and comprehensible communication style can directly influence the well-being of both the patient and the caregiver.^[22]^ Studies have shown that when physicians simplify complex medical information, it helps patients and caregivers better understand the situation, which in turn reduces anxiety and strengthens their coping capacity. Although our study did not directly examine the relationship between caregiver burden and physician communication, the observed association between higher overall perceived access to family physicians and lower caregiver burden may partly be explained by the communication and supportive functions of primary care. Easy access to the family physician not only enables medical consultation but also allows caregivers to express themselves, receive emotional support, and feel that they are not alone in the process. In this context, training programs and continuous professional development activities aimed at enhancing family physicians’ communication skills may have an indirect effect in reducing caregiver burden.

The inverse relationship found in our study between overall perceived access to family physicians and caregiver burden aligns with previous literature emphasizing the importance of social and professional support in alleviating caregiver strain. Indeed, Biney et al reported that caregivers of terminally ill cancer patients experience substantial physical, psychosocial, and economic challenges; however, religious beliefs, family ties, and support from healthcare professionals play a critical role in reducing this burden.^[4]^ However, the number of studies specifically examining the unique role of family physicians among healthcare professionals remains quite limited in the literature. In fact, a 2025 review by Lapa et al, which included 8 studies, highlighted that family physicians play a crucial role in reducing caregiver burden through providing psychological support, communicating disease processes in an understandable manner, and coordinating multidisciplinary networks.^[11]^ In the study conducted by Warner et al, it was demonstrated that primary care physicians improve outcomes for both patients and caregivers not only through clinical treatment but also by engaging in case management, assessing individual needs, and developing care plans that reflect patients’ values.^[23]^ In particular, the communication skills of primary care physicians and the timely initiation of end-of-life care planning have been reported to be critical in alleviating caregiver burden.^[23]^ Johnson et al emphasized that the accessibility of family physicians, their communication skills, and the establishment of a therapeutic relationship are fundamental elements in alleviating caregiver burden in Australia.^[22]^ Our findings support this theoretical framework with quantitative data within the Turkish context and fill an important gap in the existing literature. Notably, none of the studies included in the current review were conducted in Turkey, underscoring the clear need for local research in this area. In this respect, our research provides an evidence-based and contextually grounded contribution to the development of primary care-based palliative care practices in Turkey, while also enriching the international literature through locally derived data.

Interventional studies aimed at reducing caregiver burden have also gained increasing importance in the literature. In recent years, interventions targeting both the patient and the caregiver simultaneously have come to the forefront. In a systematic review conducted by Botas et al, the results of 8 studies were analyzed, revealing that holistic approaches such as weekly face-to-face meetings, telemedicine follow-ups, physical exercise, music therapy, and laughter therapy significantly reduced anxiety and depression levels, improved quality of life, and led to meaningful decreases in caregiver burden scores.^[24]^ Arias-Rojas et al demonstrated that a nursing-based intervention significantly improved role adaptation, perceived social support, and quality of life among family caregivers of cancer patients receiving palliative care. These findings highlight that in reducing caregiver burden, not only risk factors but also supportive and empowering interventions play a critical role.^[25]^ In addition, interventions by family physicians targeting caregivers play an important role in reducing their burden. A randomized controlled trial by Aubin et al demonstrated that emotional support provided by family physicians and their participation in regular follow-up processes significantly reduced caregivers’ stress levels.^[26]^ Similarly, Tan et al emphasized that the trust relationship and effective communication between family physicians and caregivers are among the key elements that enhance the quality of palliative care.^[27]^

This study has several limitations. First, due to its cross-sectional design, the findings indicate the direction and strength of existing associations rather than causal relationships between variables. The data were collected from a single palliative care unit, which may not fully represent care models in different institutions or regions. In addition, the use of self-reported scales carries the risk of response bias influenced by participants’ subjective perceptions. Furthermore, while excluding caregivers with chronic or psychiatric disorders enhanced sample homogeneity, it may have partially limited the generalizability of the results. Moreover, the single-center setting, together with the relatively strict exclusion criteria, may have reduced the representativeness of the sample in relation to the broader caregiver population encountered in routine palliative care practice. Another limitation is that, although the wording of the PAHCQ was adapted to specifically refer to family physicians, no separate pilot testing or psychometric validation was performed after this linguistic modification. Therefore, the possibility that this adaptation may have affected the measurement properties of the instrument cannot be completely excluded. Despite these limitations, the study has notable strengths. To our knowledge, it is one of the first quantitative investigations in Turkey to examine the relationship between perceived access to family physicians and caregiver burden during the palliative care process, providing a valuable contribution to the national literature. By exploring the interaction between primary and palliative care through quantitative data, this study offers an original and contextually grounded contribution not only to the local healthcare system but also to the evolving international literature on primary care-based palliative care integration. Future studies using multicenter and longitudinal designs may help validate the present findings. In addition, research conducted in different sociocultural contexts is expected to allow for meaningful comparisons of results. Furthermore, examining the potential associations between caregiver burden and support or communication-based approaches targeting family physicians may contribute to the advancement of knowledge in this field.

## 5. Conclusion

This study examined the relationship between caregiver burden and perceived access to family physicians among primary caregivers of terminal-stage cancer patients receiving palliative care. The findings revealed that caregiver burden increased significantly with longer daily caregiving hours and total caregiving duration, whereas higher overall perceived access to family physicians was associated with lower caregiver burden. Among the dimensions of perceived access, affordability was independently associated with lower caregiver burden. In conclusion, improving access to primary healthcare services and strengthening their connection with palliative care may enhance the quality of palliative care delivery. In this context, comprehensive national and international strategies should be developed to encourage family physicians to take a more active role in the palliative care continuum. Future multicenter and cross-cultural comparative studies conducted in different countries will help expand the global evidence base for strengthening palliative care at the primary care level.

## Acknowledgments

The author thanks statistician Nazmiye Kursun for her support in the statistical analysis of the study.

## Author contributions

**Conceptualization:** Emre Vuraloglu.

**Data curation:** Emre Vuraloglu.

**Formal analysis:** Emre Vuraloglu.

**Investigation:** Emre Vuraloglu.

**Methodology:** Emre Vuraloglu.

**Project administration:** Emre Vuraloglu.

**Resources:** Emre Vuraloglu.

**Visualization:** Emre Vuraloglu.

**Writing – original draft:** Emre Vuraloglu.

**Writing – review & editing:** Emre Vuraloglu.
